# Recurrent Steroid‐Responsive Exophthalmos as a Paraneoplastic Manifestation of Esophageal Adenocarcinoma

**DOI:** 10.1155/crom/5230454

**Published:** 2026-05-06

**Authors:** Yara E. Tovar, Ahmed G. Elsayed

**Affiliations:** ^1^ Endocrinology Department, University of Toledo, Toledo, Ohio, USA, utoledo.edu; ^2^ Hematology/Oncology Department, ProMedica Cancer Institute, Toledo, Ohio, USA

## Abstract

Exophthalmos is most often associated with autoimmune thyroid disease, particularly Graves disease. Paraneoplastic syndromes rarely manifest as orbital inflammation, and only a few cases have been described in association with solid tumors. We present a case of a 67‐year‐old male diagnosed with esophageal cancer. Shortly after diagnosis, he developed rapidly progressive bilateral exophthalmos with ophthalmoplegia. Thyroid function and thyroid‐stimulating immunoglobulin were normal, and MRI orbits demonstrated enlargement of extraocular muscles with apical crowding, consistent with inflammatory orbitopathy. Given the absence of thyroid disease, a paraneoplastic process was suspected. The patient was treated with prednisone 60 mg daily, tapered over 5 weeks, resulting in complete resolution of symptoms within 2 weeks. He underwent chemoradiation with interval improvement in the primary tumor. Several months later, disease progression occurred with new mediastinal and iliac lymphadenopathy, coinciding with recurrence of exophthalmos. A second course of corticosteroids again resulted in full remission of the orbital findings. This case highlights paraneoplastic exophthalmos as a rare manifestation of esophageal adenocarcinoma. The temporal association between tumor activity and orbital inflammation, coupled with steroid responsiveness, supports an immune‐mediated mechanism. Recognition of this phenomenon is important to avoid misdiagnosis and to guide prompt corticosteroid therapy and oncologic management. In summary, exophthalmos is a very rare paraneoplastic finding. Workup needs to include brain imaging to exclude direct metastasis to the retro‐orbital space. Immediate treatment for neoplastic disease is likely to resolve symptoms. High‐dose steroids are effective in relieving symptoms.

## 1. Introduction

Exophthalmos is a hallmark of Graves′ orbitopathy and typically occurs in the setting of autoimmune hyperthyroidism. However, similar orbital inflammatory changes may rarely arise as a paraneoplastic phenomenon secondary to systemic malignancy. Paraneoplastic orbital syndromes represent immune‐mediated reactions triggered by tumor‐associated antigens and can mimic thyroid eye disease, often leading to diagnostic uncertainty. Reports of this presentation remain exceedingly uncommon, particularly in association with gastrointestinal malignancies. We describe a patient with distal esophageal adenocarcinoma who developed recurrent, steroid‐responsive exophthalmos temporally linked to disease activity, illustrating an unusual manifestation of paraneoplastic orbital inflammation.

## 2. Case

**Figure 1:**
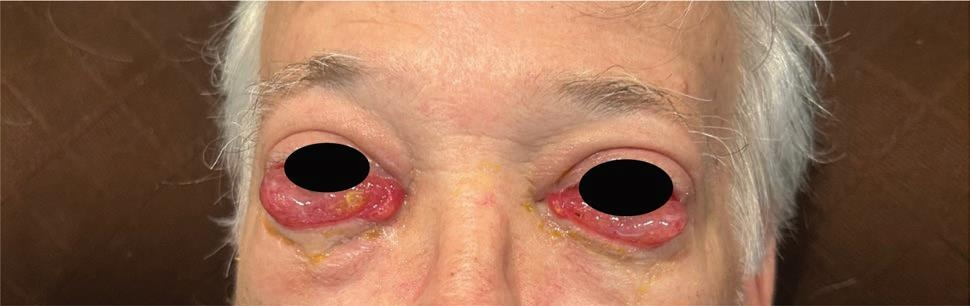
Paraneoplastic exophthalmos.

67‐year‐old male with history of hypertension, hypothyroidism, chronic kidney disease, viral cardiomyopathy, tobacco abuse. He presented with dysphagia and was found to have fungating distal esophageal mass on 05/2024. Pathology showed infiltrating moderately differentiated adenocarcinoma. PET‐CT scan showed uptake in the distal 1/3 of the esophagus with adjacent mass to the upper esophagus measuring 1.4 cm, possibly a lymph node. The scan also showed left kidney mass suspicious for malignancy. He was found to have severe exophthalmos that progressively got worse since presentation (Figure 1). An extensive workup was done including MRI orbit that confirmed the presence of exophthalmos. The thyroid workup was unremarkable including normal TSH and free T4: TSH 1.54 uIU/mL, freeT4 = 0.79 ng/dL, and undetectable thyroid stimulating immunoglobulin.

**Figure 2:**
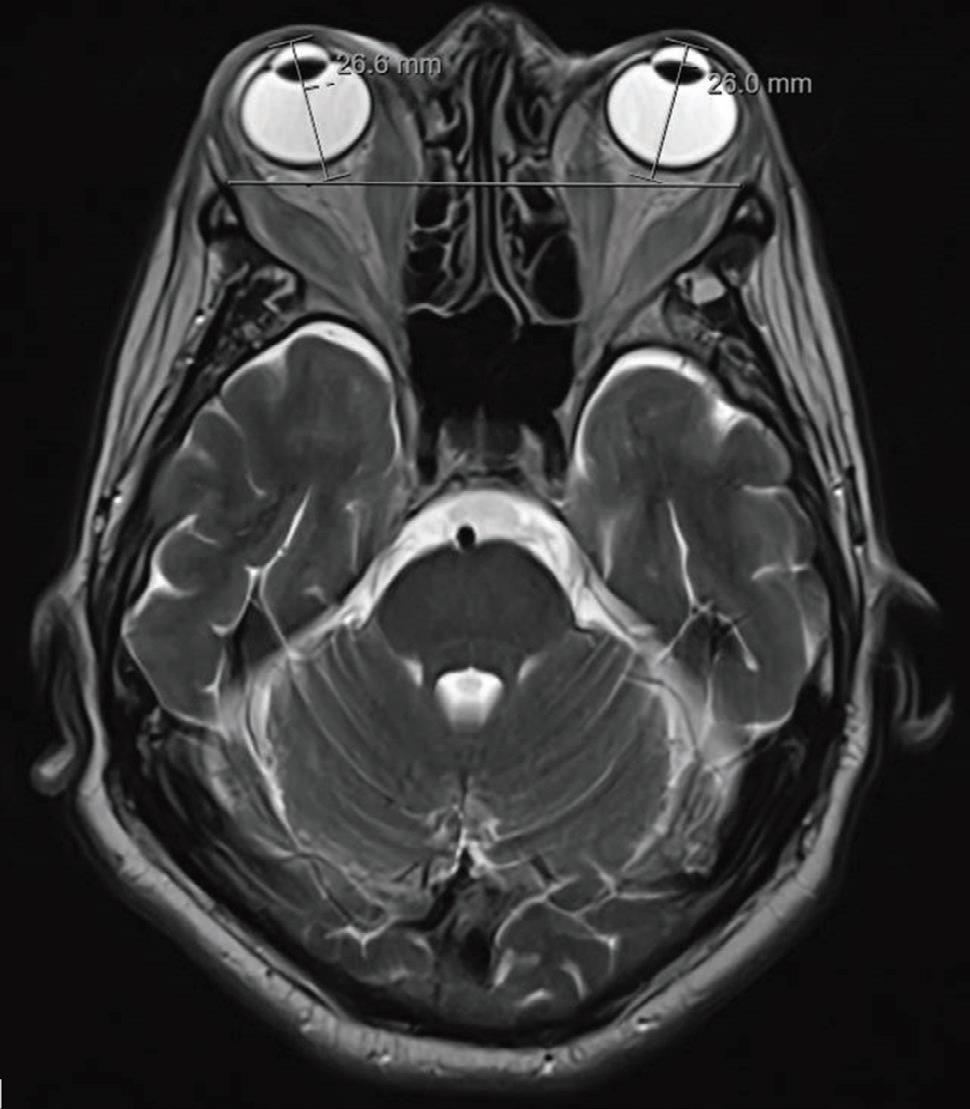
MRI showing inflammatory orbitopathy causing exophthalmos.

Physical exam by ophthalmology revealed complete ophthalmoplegia, bilateral upper lid retraction with temporal flare, bilateral lower lid retraction, and evidence of exposure issues with prolapsing and keratinized chemosis inferiorly. The cornea was unremarkable. The conjunctiva did not have corkscrew vessels. Orbital ultrasound showed low reflectivity and enlarged extraocular muscles. Orbital MRI showed expansion of the orbital fat compartments, enlargement of the inferior rectus, medial rectus, superior rectus, and lateral rectus bilaterally, with sparing of the muscle tendons anteriorly but involved posteriorly (Figure 2).

A paraneoplastic panel was sent, and that was negative. This included anti‐N‐methyl‐D‐aspartate receptor antibodies, antiglial neuronal nuclear autoantibody‐Type 1, antineuronal nuclear antibody Type 2, amphiphysin antibody, and anticontactin‐associated protein‐2 (CASPR2).

**Figure 3:**
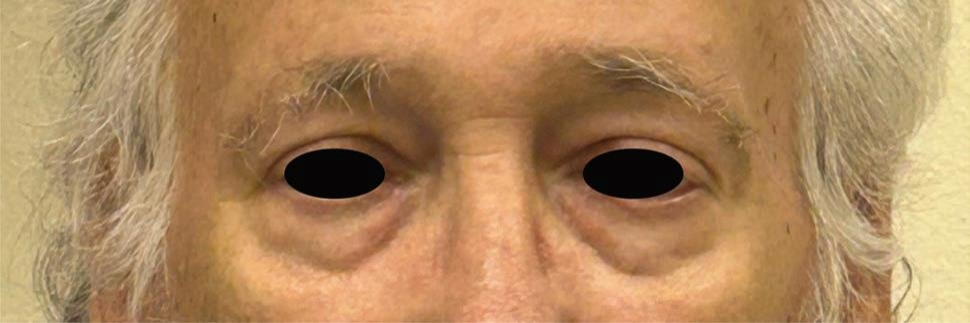
Complete resolution with steroids.

Eventually, this was felt to be a paraneoplastic syndrome. The patient was started on prednisone 60 mg, which was weaned off over the course of 5 weeks. The patient had remarkable improvement with the steroids, with complete resolution of his exophthalmos after 2 weeks of treatment (Figure 3).

Subsequently, the patient was treated with neoadjuvant concurrent chemoradiation with carboplatin and paclitaxel as per the Cross trial regimen [1], starting on 07/2024 and completed in 09/2024. Posttreatment PET‐CT scan done 10/2024 showed a response to treatment with decreased metabolic activity in the known neoplasm involving the distal 3rd of the esophagus and gastric fundus. The left paraoesophageal lymph node decreased in size and activity. The left upper pole kidney lesion increased in size and metabolic activity. This was biopsied on 11/2024 and showed renal cell cancer. The patient was deemed to be at high risk for esophagectomy because of his underlying cardiomyopathy. He opted for radiological surveillance.

PET‐CT scan done in 02/2025 showed radiological evidence of disease progression with new PET‐avid lymph nodes in the iliac region, right tracheobronchial, mediastinum, and other smaller periesophageal lymph nodes. Biopsy of the left external iliac chain lymph node confirmed the recurrence of esophageal cancer. Interestingly, the patient started noticing the relapse of his exophthalmos at the same time he had relapsed esophageal cancer. The exophthalmos started to get worse progressively until it became as severe as it was prior to treatment. He responded again to prednisone 60 mg, which was weaned off over the course of 5 weeks, with complete resolution of the exophthalmos. The patient was started on palliative systemic chemotherapy, but eventually his performance status dropped, and he opted to go to hospice. He expired shortly afterwards.

## 3. Discussion

Exophthalmos is usually a finding associated with hyperthyroid status. It is well described in this clinical context. Treatment of the hyperthyroid status may help improve eye symptoms, but does not usually lead to complete resolution [2]. Paraneoplastic syndromes are a group of clinical symptoms that occur in the setting of malignancy and are not directly related to it. Common paraneoplastic syndromes include hypercalcemia, neuropathy, and Lambert–Eaton myasthenic syndrome. Paraneoplastic syndromes presenting with neuro‐ophthalmologic symptoms are rare. Some examples of those include cancer‐associated retinopathy, Uveal proliferation, and paraneoplastic optic neuropathy [3–8].

Paraneoplastic proptosis is a rare occurrence. Few cases were reported in the literature. Yoshida et al. reported its occurrence with early‐stage lung cancer with resolution after resection [8]. A similar case with bilateral proptosis associated with large cell bronchial carcinoma was reported. It was completely resolved with resection of the lung tumor [3]. Romano et al. reported another case with paraneoplastic proptosis with lung cancer [9]. The earliest case report identified in the literature search was reported by Harris et al. [10]. Proptosis was associated with non‐Hodgkin lymphoma. Biopsy showed orbital myositis without lymphomatous involvement. Proptosis improved with immunosuppressive therapy despite lymphoma progression [10].

A suggested pathophysiology for this finding is an immune‐mediated inflammatory reaction within the orbital muscles, leading to swelling and subsequent exophthalmos. There is no standard treatment for this condition beyond treating the underlying malignancy, as is the case with all paraneoplastic syndromes. The case we presented, as well as some other cases presented in the literature, responded very well to steroids [4].

It is important to mention that the exophthalmos in our case remained well controlled as long as the patient′s esophageal cancer was controlled. Exophthalmos reoccurred in our case with relapse of the disease and responded again to the steroids. Solid tumors do not usually respond immediately to chemotherapy, and for that reason, we opted to proceed with steroids until adequate control of the neoplastic process is achieved.

## 4. Conclusion

Paraneoplastic orbital inflammation should be considered in patients presenting with exophthalmos and normal thyroid function, particularly when symptoms parallel malignancy activity. Corticosteroids can be highly effective in symptom control, but long‐term outcomes depend on tumor response.

## Funding

No funding was received for this manuscript.

## Consent

Patient signed a written informed consent and agreed to publish photos provided in the case.

## Conflicts of Interest

The authors declare no conflicts of interest.

## Data Availability

Data sharing not applicable to this article as no datasets were generated or analyzed during the current study.
